# Long-Term Impact of a Smartphone App on Prescriber Adherence to Antibiotic Guidelines for Adult Patients With Community-Acquired Pneumonia: Interrupted Time-Series Study

**DOI:** 10.2196/42978

**Published:** 2023-05-02

**Authors:** Chang Ho Yoon, Imogen Nolan, Gayl Humphrey, Eamon J Duffy, Mark G Thomas, Stephen R Ritchie

**Affiliations:** 1 Big Data Institute Oxford United Kingdom; 2 Infectious Diseases Department Auckland City Hospital Auckland New Zealand; 3 National Institute for Health Innovation University of Auckland Auckland New Zealand; 4 School of Medical Sciences University of Auckland Auckland New Zealand

**Keywords:** app, antimicrobial stewardship, antibiotic adherence, community, pneumonia, smartphone, mobile health, mHealth, antibiotic, behavior, adults, diagnosis, pulmonary, patient

## Abstract

**Background:**

Mobile health platforms like smartphone apps that provide clinical guidelines are ubiquitous, yet their long-term impact on guideline adherence remains unclear. In 2016, an antibiotic guidelines app, called SCRIPT, was introduced in Auckland City Hospital, New Zealand, to provide local antibiotic guidelines to clinicians on their smartphones.

**Objective:**

We aimed to assess whether the provision of antibiotic guidelines in a smartphone app resulted in sustained changes in antibiotic guideline adherence by prescribers.

**Methods:**

We analyzed antibiotic guideline adherence rates during the first 24 hours of hospital admission in adults diagnosed with community-acquired pneumonia using an interrupted time-series study with 3 distinct periods post app implementation (ie, 3, 12, and 24 months).

**Results:**

Adherence increased from 23% (46/200) at baseline to 31% (73/237) at 3 months and 34% (69/200) at 12 months, reducing to 31% (62/200) at 24 months post app implementation (*P*=.07 vs baseline). However, increased adherence was sustained in patients with pulmonary consolidation on x-ray (9/63, 14% at baseline; 23/77, 30% after 3 months; 32/92, 35% after 12 month; and 32/102, 31% after 24 months; *P*=.04 vs baseline).

**Conclusions:**

An antibiotic guidelines app increased overall adherence, but this was not sustained. In patients with pulmonary consolidation, the increased adherence was sustained.

## Introduction

Antibiotic stewardship programs in hospitals and community clinics strive to improve rates of appropriate antibiotic prescribing through a wide variety of methods (from clinical decision support tools to educational sessions) both to optimize the treatment of patients with bacterial infections and to reduce inappropriate antibiotic prescribing [1]. Greater adherence to antibiotic guidelines (ie, prescription of antibiotics consistent with guidelines) is associated with better treatment outcomes and reduced antibiotic resistance [1,2], yet rates of adherence remain low [3-7]. Despite the ubiquity and promise of mobile health (mHealth) platforms like smartphone apps to overcome some of the causes of low adherence, such as limited access to guidelines, the long-term impact of mHealth apps on guideline adherence remains unclear [8].

**Figure 1:**
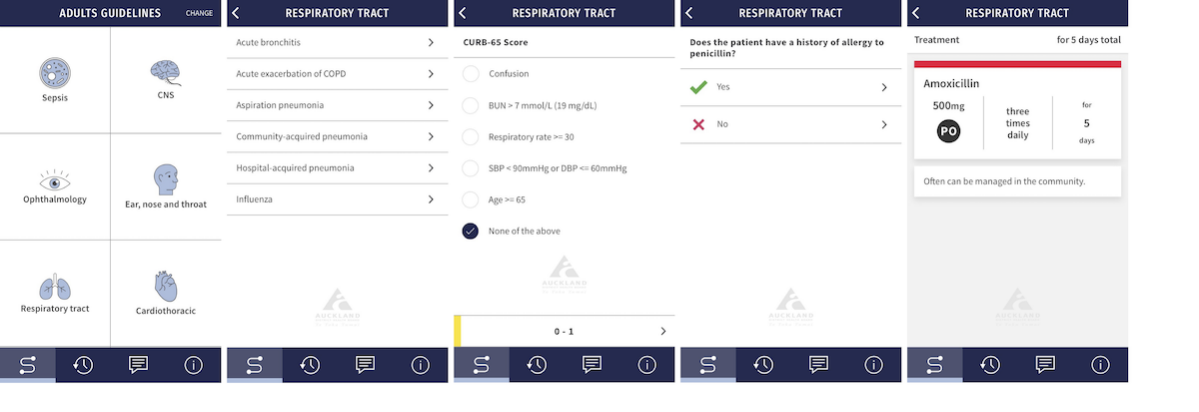
Succession of screenshots (left to right) from the SCRIPT smartphone app, which displays the user interface in accessing antibiotic guidelines for low-risk community-acquired pneumonia as defined by a CURB-65 score of 0-1 (middle screenshot).

A small number of studies have suggested that apps displaying antibiotic guidelines improve antibiotic prescribing behavior in the short term [8], while the only study to have measured adherence beyond 12 months post app implementation suggested that any improvements are not necessarily sustained [9]. Therefore, the long-term influence of such apps requires further investigation, with implications for their cost-benefit analysis and long-term utility in antibiotic stewardship programs. In 2016, we developed an antibiotic guidelines app, “SCRIPT,” at Auckland City Hospital (ACH) in New Zealand, which displayed antibiotic guidelines for community-acquired pneumonia (CAP) and urinary tract infection to prescribing clinicians on their smartphones and observed improvement in adherence for patients with CAP but not for patients with urinary tract infection [6]. However, this study was limited by only 3 months of follow-up and a short lead-in time for app adoption. Since then, SCRIPT has increased in popularity among local prescribers and become a standard part of the prescribing repertoire, evidenced by 5600 unique users accounting for 600,000 app sessions in 2020 and over 700,000 in 2021 [10]. The app is freely available (as “SCRIPT ADHB” via Google Play and App Store) and sports a simple, user-friendly interface (Figure 1). Given SCRIPT’s widespread adoption in ACH, we aimed to assess its long-term impact on prescriber adherence to antibiotic guidelines in patients with CAP at 3, 12, and 24 months after the SCRIPT guidelines were made available.

## Methods

### Aims and Study Setting

We performed an interrupted time-series study to test the hypothesis that the provision of the SCRIPT app would increase prescriber adherence to antibiotic guidelines for hospitalized adult patients with CAP. We further hypothesized that adherence to antibiotic guidelines would be higher in cases with chest x-ray evidence of CAP than in cases without chest x-ray evidence of CAP (because the diagnosis of CAP is questionable in cases without chest x-ray evidence [11,12]). The initial impact of SCRIPT’s implementation on adherence to local ACH antibiotic guidelines has been described previously [6]. In the first 2 weeks of the intervention period, educational sessions, posters, and intranet advertisements were employed to socialize the app and facilitate its uptake by ACH clinicians. Thereafter, the app was promoted periodically in newsletters and posters and at each orientation for new rotations of junior doctors.

ACH has a multifaceted approach to antimicrobial stewardship (including formulary management, regular audit and feedback, expert consultation services, and surveillance of antibiotic use), which continued unaltered throughout the study period. The hospital antibiotic guidelines remained on the hospital intranet. No other interventions impacting CAP management were introduced during the study period.

### Study Cohort

We retrospectively collected data during 4 periods, as follows: “baseline” pre-app implementation (January 1 to May 31, 2016); “immediate” post-app implementation (June 1 to August 31, 2016); 12-month post-app implementation (June 1 to October 31, 2017); and 24-month post-app implementation (June 1 to October 31, 2018).

Adult patients (aged ≥18 years) admitted to ACH for ≥4 hours with a discharge diagnosis of CAP (International Classification of Diseases-10 codes: J10-18 and J22) were included. Patients were excluded if they were not diagnosed with CAP during the first 24 hours of admission; had incorrectly coded diagnoses (eg, “empyema”); or were transferred from another secondary or tertiary care facility where antibiotics had been administered.

All CAP cases during each period were identified. We used Microsoft Excel’s random number generator to randomly select ≥200 cases per period (200 at “baseline”; 237 in the “immediate” post-app period; 200 at 12 months; and 200 at 24 months). We calculated that inclusion of these case numbers would achieve 90% power to detect an absolute 15% increase in guideline adherence (=.05) [6].

All patients had a chest x-ray at admission to detect radiological features of consolidation, defined as 1 or more opacities in the lung fields consistent with the diagnosis of pneumonia.

### Data Collection and Definitions

Electronic health record data were collected using REDCap (version 6.5.15; Vanderbilt University) to record demographic (eg, age, sex, and ethnicity) and clinical data (eg, admission date; diagnostic impression at admission; vital signs at admission—documentation of confusion in the patient, respiratory rate, systolic and diastolic blood pressures; urea; and presence of consolidation on chest x-ray at admission, as reported by a radiologist) as well as antibiotics prescribed (eg, drug name, route, and duration) during the first 24 hours post admission.

Adherence was defined as prescription of antibiotic(s), including dose(s) and route(s) of administration, according to local guidelines, during the first 24 hours post admission. The ACH antibiotic guidelines for CAP vary by the CURB-65 pneumonia severity score, where a point is given for each of the prognostic features (C: confusion, U: increased serum urea concentration, R: respiratory rate ≥30 breaths/min, B: systolic blood pressure <90 mmHg or diastolic blood pressure ≤60 mmHg, and 65: age ≥65 years). Cases with a total CURB-65 score of 0-1 were considered to be at low risk (<10%) of mortality; those with a score of 2 were at intermediate risk (10%-20%) of mortality; and a score of 3-5 indicated high risk (20%-60%) of mortality [13]. In cases whose serum urea concentration had not been measured, CRB-65 scores were calculated. CRB-65 is a validated alternative to CURB-65, shown to be predictive of mortality in patients hospitalized with pneumonia [14]. A CRB-65 score of 0 would equate to a CURB-65 score of 0 at best and 1 at worst; thus, we elected to use CURB-65 score ranges (0-1, 1-2, 2-3, and 3-5). If the attending clinicians had not documented the CURB-65 score in the clinical records, we calculated the patient’s CURB-65 score using relevant data available to the clinicians when selecting antibiotic management. Antibiotic guideline adherence was assessed according to the actual or highest possible CURB-65 score.

Other antibiotic(s), prescribed in addition to guideline-adherent antibiotic(s), were considered unnecessary additional antibiotics. Undertreatment was defined as prescription of an inappropriately narrow-spectrum regimen (eg, prescription of amoxicillin alone for severe CAP).

These definitions were applied by 2 physicians (CHY and SRR) and an infectious diseases specialist pharmacist (EJD) based on the assumption that the patient had CAP, regardless of the presence of pulmonary consolidation on chest x-ray (a defining characteristic of CAP, the absence of which does not preclude the diagnosis of CAP) [15].

### Analysis

Statistical analyses were performed using R (version 4.0.3; The R Core Team). Rates of adherence, use of unnecessary additional antibiotics, and undertreatment were compared between study periods and between cases with or without pulmonary consolidation on the admission chest x-ray (based on the reporting radiologist’s assessment), using Pearson chi-square test or Fisher exact test (significance level: α=.05). One case, in the immediate follow-up group, did not have a chest x-ray and was excluded from analyses that compared patients with or without pulmonary consolidation.

### Ethics Approval

All analyses were performed in accordance with the study protocol for which ethics approval was granted (New Zealand Health and Disabilities Ethics Committee reference number: 16/STH/6).

## Results

### Demographic And Clinical Features

The sex, median ages, and ethnicities of the patients in the 4 cohorts were broadly similar (Table 1). The proportions of patients with consolidation on chest x-ray (an initial diagnostic impression of pneumonia) and prescriber-documented CURB-65 scores were higher in the 12-month and 24-month cohorts compared to the baseline cohort. In all 4 cohorts, most patients with consolidation on chest x-ray (43/63, 68% at baseline; 54/77, 70% in the immediate post-app period; 65/92, 71% at 12 months; and 69/102, 68% at 24 months) had an initial diagnostic impression of “pneumonia.” By contrast, in all 4 cohorts, a minority of patients without consolidation on chest x-ray (25/137, 18% at baseline; 14/159, 9% in the immediate post-app period; 31/108, 29% at 12 months; 24/98, 24% at 24 months) had an initial diagnostic impression of “pneumonia” (*P*<.001).

**Table 1:** Demographic and clinical features and overall adherence to antibiotic guidelines for patients with community-acquired pneumonia admitted to Auckland City Hospital in the baseline, immediate, 12-month, and 24-month cohorts.

Cohort			Baseline (n=200)		Immediate (n=237)		12-month (n=200)		24-month (n=200)
Age (years), median (IQR)			62 (46-77)		64 (44-79)		70 (53-82)		67 (51-80)
**Sex, n (%)**									
	Female	96 (48)		139 (59)		94 (47)		112 (56)	
	Male	104 (52)		98 (41)		106 (53)		88 (44)	
**Ethnicity, n (%)**									
	Asian or other	47 (24)		32 (14)		41 (20)		38 (19)	
	Māori	15 (7.5)		29 (12)		14 (7)		21 (10)	
	New Zealand European	91 (46)		121 (51)		101 (50)		95 (48)	
	Pacific	47 (24)		55 (23)		44 (22)		46 (23)	
Chest x-ray consolidation, n (%)			63 (32)		77 (33)		92 (46)		102 (51)
**Initial diagnostic impression n (%)**									
	Pneumonia	68 (34)		68 (29)		96 (48)		93 (46)	
	Lower respiratory tract infections (unspecified)	103 (52)		97 (41)		59 (30)		59 (30)	
	Viral illness	15 (7.5)		61 (26)		27 (14)		28 (14)	
	Bronchitis or other	14 (7)		11 (4.6)		18 (9)		20 (10)	
**CURB-65 score estimated from clinical data, n (%)**									
	0-1	87 (44)		102 (43)		62 (31)		68 (34)	
	1-2	68 (34)		95 (40)		84 (42)		70 (35)	
	2-3	40 (20)		34 (14)		39 (20)		51 (26)	
	3-5	5 (2.5)		6 (2.5)		15 (7.5)		11 (5.5)	
Length of stay (days), median (IQR)			2.0 (1.0-4.0)		2.0 (1.0-4.0)		2.0 (1.0-5.0)		2.0 (1.0-4.2)
Adherence to antibiotic guidelines, n (%)			46 (23)		73 (31)		69 (34)		62 (31)

### Overall Antibiotic Guideline Adherence

Compared with the baseline cohort (46/200, 23%), there was a nonsignificant increase in prescriber adherence to the antibiotic guideline in the immediate cohort (73/237, 31%) but a significant increase in adherence in the 12-month cohort (69/200, 34%; *P*=.01), which was not sustained in the 24-month cohort (62/200, 31%; Table 1).

### Antibiotic Guideline Adherence in Patients With Pulmonary Consolidation

For patients with consolidation on chest x-ray, antibiotic guideline adherence increased from 14% (9/63) in the baseline cohort to 30% (23/77) in the immediate cohort—a change that was sustained in the 12-month cohort (32/92, 35%) and in the 24-month cohort (32/102, 31%; *P*=.04; Table 2). There were no significant differences between cohorts in the prescription of unnecessary additional antibiotics or in undertreatment.

**Table 2:** Adherence to antibiotic guidelines, use of additional unnecessary antibiotics, undertreatment, and diagnostic features for cases with or without consolidation on admission chest x-ray (a definitive diagnosis of pneumonia requires radiographic evidence of consolidation, but the absence of consolidation does not necessarily preclude the diagnosis) [15].

Characteristics			Consolidation												No consolidation									
			Baseline (n=63), n (%)		Immediate (n=77), n (%)		12 months (n=92), n (%)		24 months (n=102), n (%)		*P* value^a^			Baseline (n=137), n (%)			Immediate (n=159), n (%)		12 months (n=108), n (%)		24 months (n=98), n (%)		*P* value^a^	
**Adherence**												.04												.67
	Adherent	9 (14)		23 (30)		32 (35)		32 (31)					37 (27)			50 (31)		37 (34)		30 (31)				
	Nonadherent	54 (86)		54 (70)		60 (65)		70 (69)					100 (73)			109 (69		71 (66)		68 (69)				
**Unnecessary additional antibiotics**												.91												.001
	No	38 (60)		50 (65)		59 (64)		62 (61)					103 (75)			107 (67)		96 (89)		70 (71)				
	Yes	25 (40)		27 (35)		33 (36)		40 (39)					34 (25)			52 (33)		12 (11)		28 (29)				
**Undertreatment**												.43												.55
	No	47 (75)		64 (83)		78 (85)		82 (80)					99 (72)			123 (77)		77 (71)		76 (78)				
	Yes	16 (25)		13 (17)		14 (15)		20 (20)					38 (28)			36 (23)		31 (29)		22 (22)				
**Initial diagnostic impression**												.18												<.001
	Pneumonia	43 (68)		54 (70)		65 (71)		69 (68)					25 (18)			14 (8.8)		31 (29)		24 (24)				
	LRTI^b^ (unspecified)	16 (25)		18 (23)		14 (15)		17 (17)					87 (64)			78 (49)		45 (42)		42 (43)				
	Viral illness	0 (0)		3 (3.9)		7 (7.6)		5 (4.9)					15 (11)			58 (36)		20 (19)		23 (23)				
	Bronchitis or other	4 (6.3)		2 (2.6)		6 (6.5)		11 (11)					10 (7.3)			9 (5.7)		12 (11)		9 (9.2)				
CURB-65^c^ score documented by prescriber			17 (27)		35 (45)		33 (36)		28 (27)		.046			15 (11)			15 (9.4)		17 (16)		16 (16)		.26	
**CURB-65 score calculated from clinical data**												.80												.002
	0-1	22 (35)		31 (40)		31 (34)		35 (34)					65 (47)			71 (45)		31 (29)		33 (34)				
	1-2	24 (38)		27 (35)		36 (39)		32 (31)					44 (32)			67 (42)		48 (44)		38 (39)				
	2-3	15 (24)		14 (18)		17 (18)		26 (25)					25 (18)			20 (13)		22 (20)		25 (26)				
	3-5	2 (3.2)		5 (6.5)		8 (8.7)		9 (8.8)					3 (2.2)			1 (0.6)		7 (6.5)		2 (2)				

^a^Chi-square test and Fisher exact test.

^b^LRTI: lower respiratory tract infection.

^c^Pneumonia severity score (C: confusion, U: increased serum urea concentration, R: respiratory rate ≥30 breaths/min, B: systolic blood pressure <90 mmHg or diastolic blood pressure ≤60 mmHg, and 65: age ≥65 years).

## Discussion

In patients with CAP and pulmonary consolidation on chest x-ray, there was a sustained improvement in guideline adherence. However, in patients with CAP without consolidation, where the most common diagnostic impression was “viral illness” or “lower respiratory tract infections (unspecified),” guideline adherence was not sustained. The sustained improvement in adherence to the guidelines for treatment of CAP in patients with consolidation on chest x-ray indicates that clinicians were adapting their use of the guideline to increase their use of it in those patients for whom they thought the guideline was most appropriate. This evolution of prescriber use of the guideline over time is an encouraging feature, particularly given the absence of other initiatives to improve prescribing for CAP, suggesting that prescribers were intellectually engaging with the guideline. An appropriate response by those responsible for maintaining and updating the guideline might be to include the presence or absence of consolidation on the chest x-ray as a decision point in the treatment algorithm.

The only other published study of the long-term impact of an antibiotic guidelines app on prescriber adherence was performed in 3 hospitals in west London, where baseline rates of adherence were high (75%-90%) [9]. The introduction of a smartphone app resulted in a significant increase in the rate of adherence for surgical patients, sustained at 24 months. However, in medical patients, a nonsignificant increase in the rate of adherence was followed by a gradual decline toward preintervention levels. In our study of medical patients with CAP, preintervention rates of adherence were low (46/200, 23%) but improved significantly to 34% (69/200) at 12 months post app implementation, before then declining to 31% (62/200). Our findings are broadly consistent with those of Charani et al [9], who found an initial increase followed by a subsequent decline in guideline adherence in medical patients. It should be noted that adherence to guidelines in our study required that the antibiotic, dose, and mode of administration be as stated in our guidelines; however, the definition used in the Charani et al [9] study required that only the antibiotic were that stated in their guidelines and did not require the dose and mode of administration to be the same as those in the guidelines.

Although very high uptake and use of the app at ACH (>1000 new downloads each year, over half of which are by junior doctors) enabled this real-world evidence study, there were no data directly matching the use of the SCRIPT app by the clinicians whose antibiotic prescriptions were analyzed in this study, that is, we were not able to measure the direct influence of using the app on individual cases of antibiotic prescription but rather the average net effect of making such an app available. Other limitations included the unmeasured impact of team-based decisions (vs individual decisions) for antibiotic prescriptions and of junior doctors changing clinical jobs every few months at ACH, moving to or from other hospitals, which would periodically and variably diminish the proportions of doctors using the app at ACH. We were not able to assess the app’s impact relative to other antibiotic stewardship methods nor to other variables that may influence guideline adherence, such as the prescriber’s level of seniority, where they had previously worked, their specialty, and patient-related factors like comorbidity and illness acuity.

A range of technological advances, including antibiotic guidelines apps and computerized decision support systems appear to offer opportunities to dramatically improve adherence to prescriber guidelines. However, as with our study, it is rare that such advances provide a silver bullet for the widespread, recalcitrant problem of low adherence to antimicrobial prescribing guidelines. Instead, it is common for such advances to provide modest improvements, commonly of a 10%-20% absolute improvement in guideline adherence, when a 30%-50% absolute increase would have been required to achieve adherence rates above 90% [16-19]. Although mHealth solutions have been perceived to be convenient and effective in improving guideline adherence, their high cost would be more justified should their impact be more long-term; this is especially pertinent in multimodal antibiotic stewardship programs, where there would be further opportunity costs.

Causes of failure to achieve large changes in antibiotic guideline adherence include within-team dynamics that may contribute to lack of support for changes in prescriber behavior. Junior clinicians, who write almost all prescriptions, may be more influenced by the entrenched opinions of their senior colleagues than by the advice contained in a guideline [20,21]. Other causes of low adherence may pertain to app-related factors like usability, acceptability, and app fatigue, although SCRIPT was designed using state-of-the-art co-design approaches through interactions between designers and end-user stakeholders [22,23]. Moreover, rates of SCRIPT use at Auckland Hospital have steadily increased rather than declining, suggesting that the app has high usability with no evidence of app fatigue. SCRIPT can only provide guidelines, not actively reinforce them. e-Prescribing may be able to address this gap and could be the subject of future studies in antibiotic guideline adherence.

Overall, our results suggest that a highly used antibiotic guidelines app can help to increase overall rates of prescriber adherence, especially in those patients with the strongest evidence that they fall into the diagnostic group the treatment advice is intended for and in those patients with more severe diseases. Sustaining increased rates of adherence likely requires refinement of the app algorithms in response to evidence that prescribers are selective in their adherence to guidelines and may respond to clinical features that are not included in the app algorithms. As with all innovations, a continuous process of development, testing, analysis, and modification is necessary to achieve the best results.

## Abbreviations

ACH: Auckland City Hospital
CAP: community-acquired pneumonia
mHealth: mobile health

## Data Availability

The data analyzed are not publicly available as they contain personal data but may be made available subject to an application and research proposal meeting the ethical and governance requirements of accessing the data.
